# Supplementary material for: Streptomyces umbrella toxin particles block hyphal growth of competing species
Source: Nature. 2024 Apr 17;629(8010):165–73. doi: 10.1038/s41586-024-07298-z (PMC11062931; doi:10.1038/s41586-024-07298-z)
Supplement: Supplementary file 1 — This file contains five supplementary figures and two supplementary tables. Figures include uncropped blots and gels, information supporting the cryo-EM structure determination of Umb1, and information related to Umb loci characterization. Tables contain raw data from IP–MS experiments and Umb toxicity screening. [file 41586_2024_7298_MOESM1_ESM.pdf]

nature portfolio

https://doi.org/10.1038/s41586-024-07298-z

Supplementary information

Streptomyces umbrella toxin particles block hyphal growth of competing species

In the format provided by the authors and unedited

Supplementary Information

Streptomyces umbrella toxin particles block hyphal growth of competing species

Qinqin Zhao, Savannah Bertolli, Young-Jun Park, Yongjun Tan, Kevin J. Cutler, Pooja Srinivas, Kyle Asfahl, Citlali F. Garcia, Larry A. Gallagher, Yaqiao Li, Yaxi Wang, Devin Coleman-Derr, Frank DiMaio, Dapeng Zhang, S. Brook Peterson, David Veesler and Joseph D. Mougous

Supplementary Figures

Uncropped blots, Fig. 2c

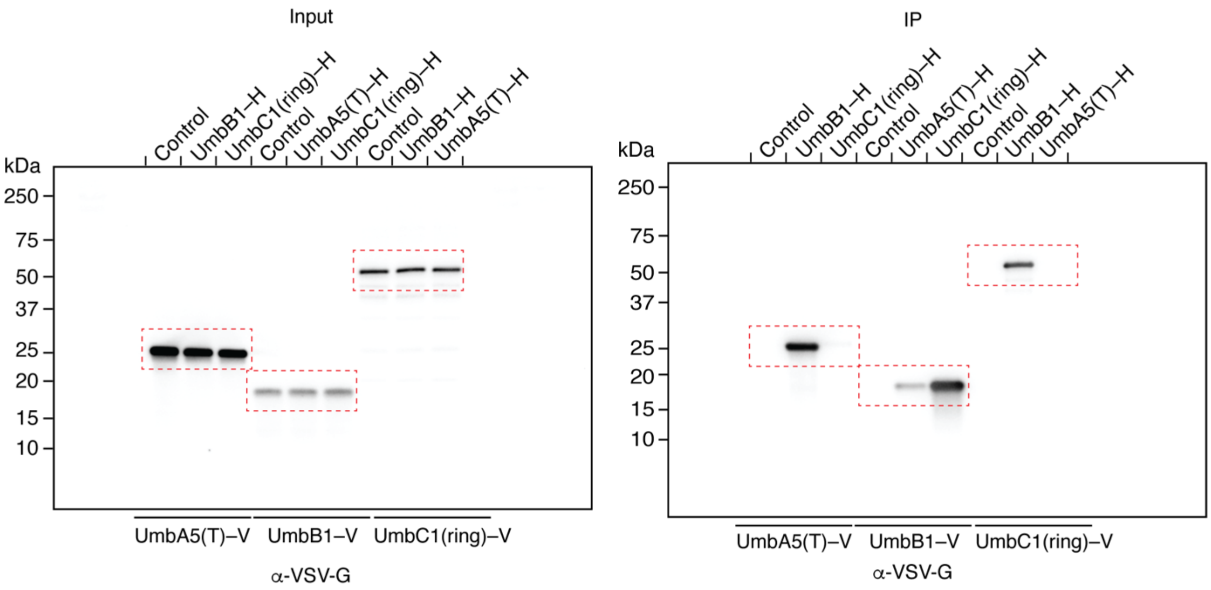

Uncropped blots, Fig. 2d

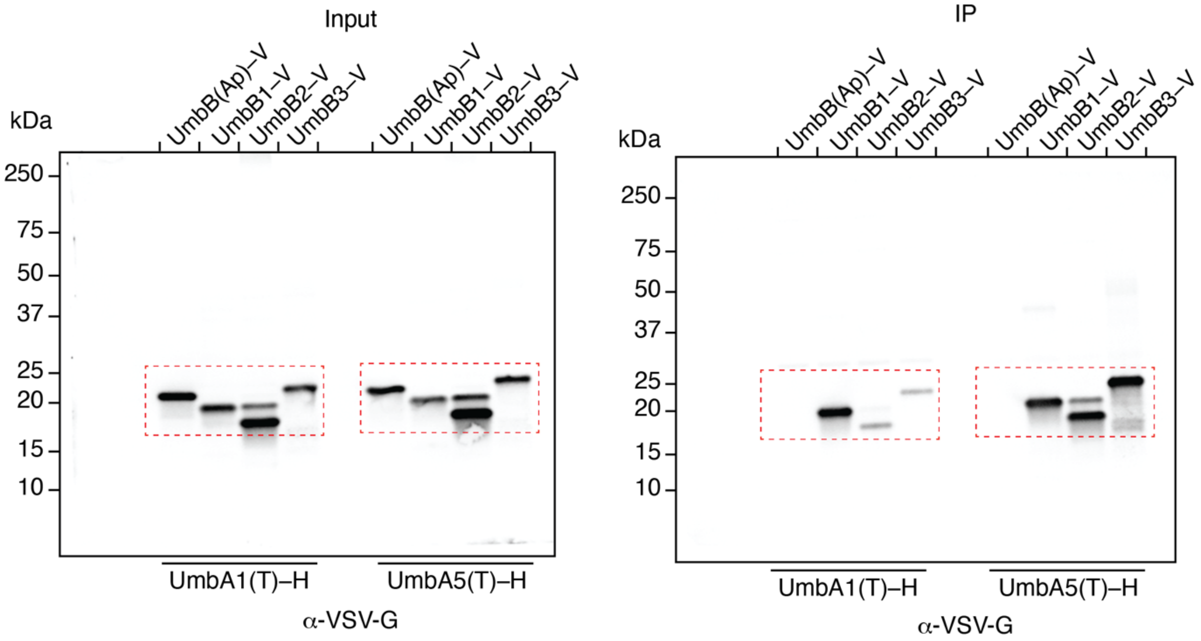

Uncropped blots, Fig. 2e

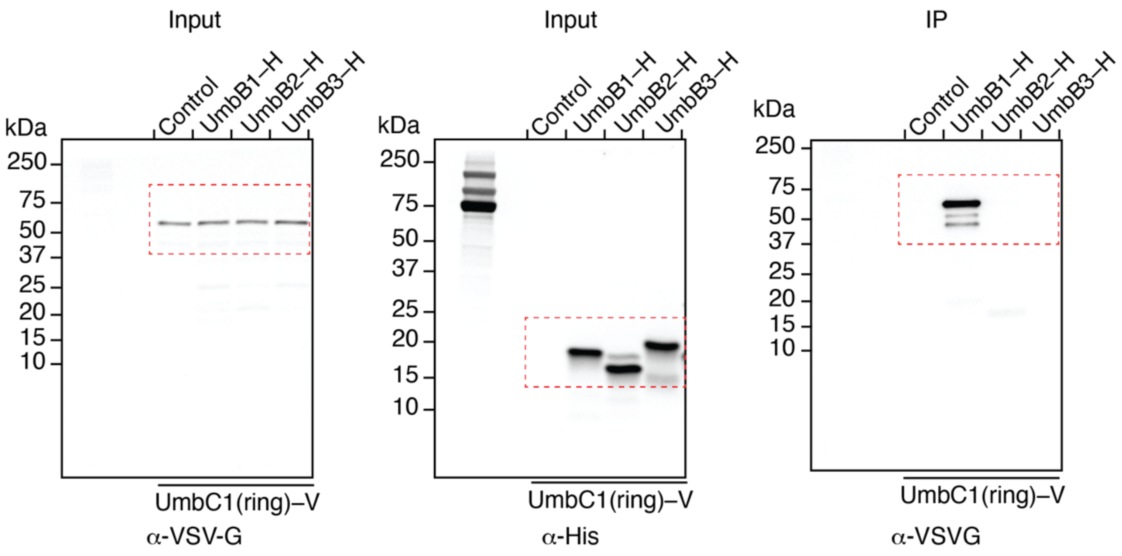

Uncropped blots, Fig. 2f

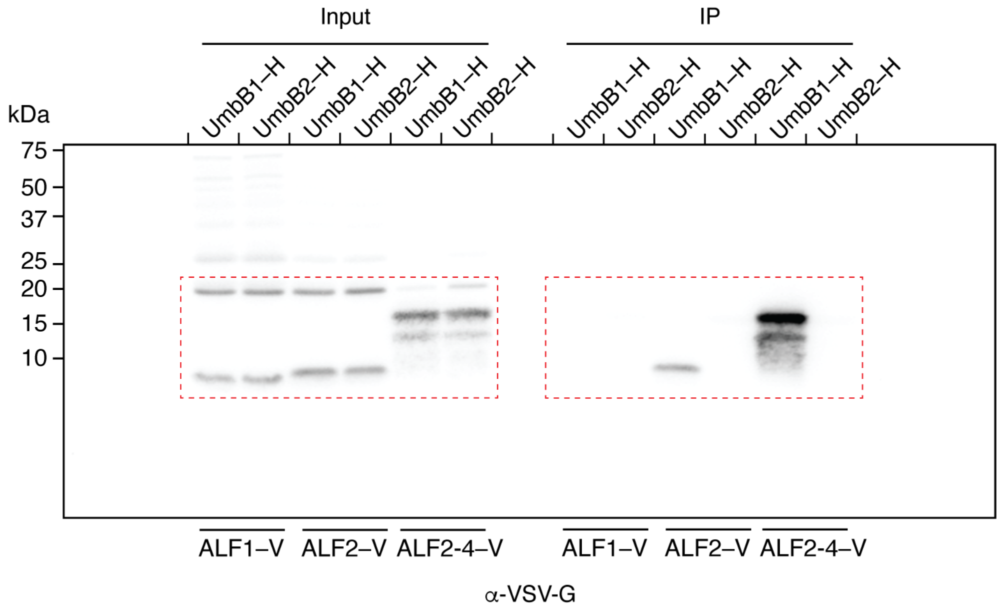

Uncropped blots, Fig. 2j and 2k

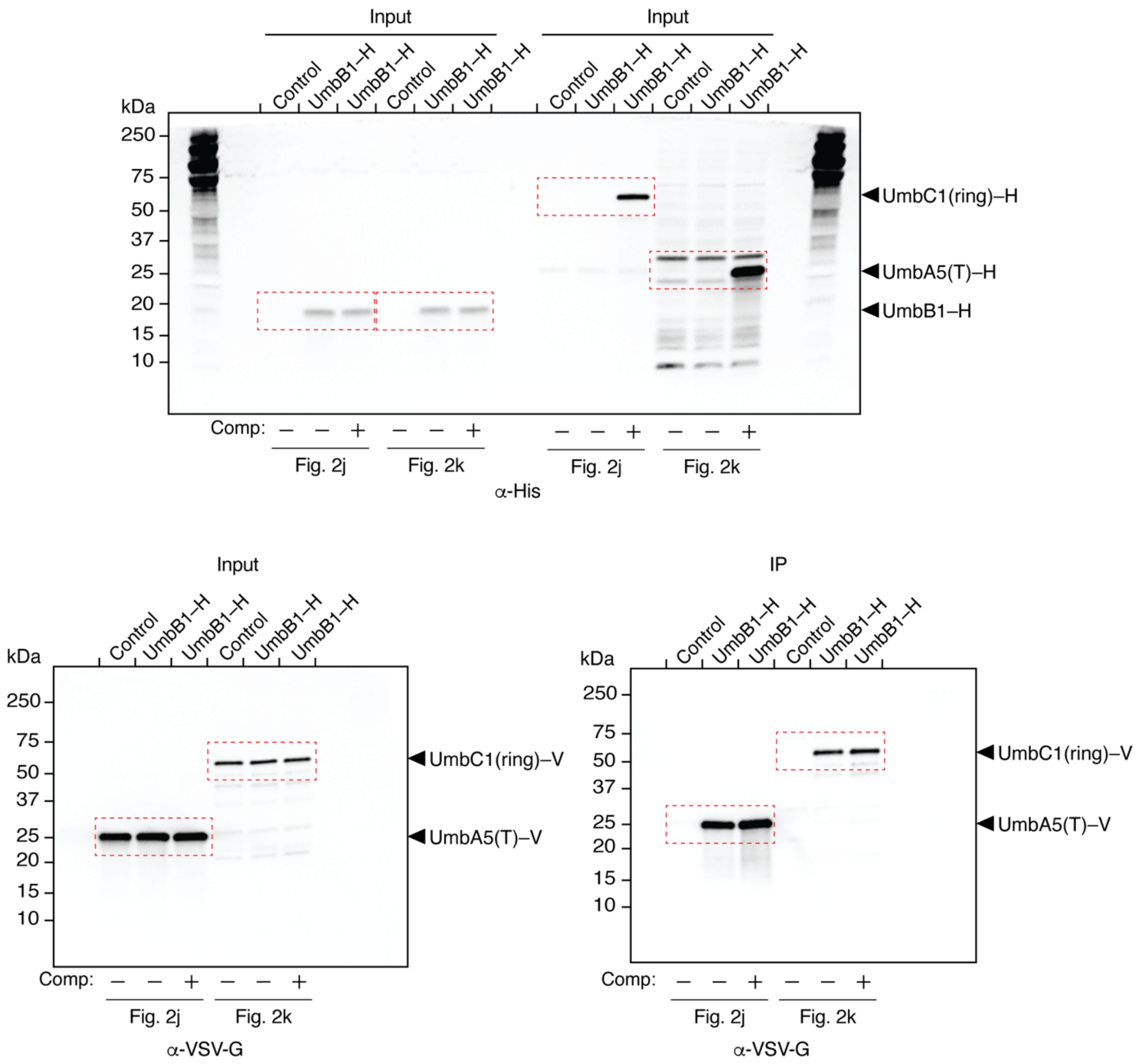

Uncropped gel, Fig. 3a

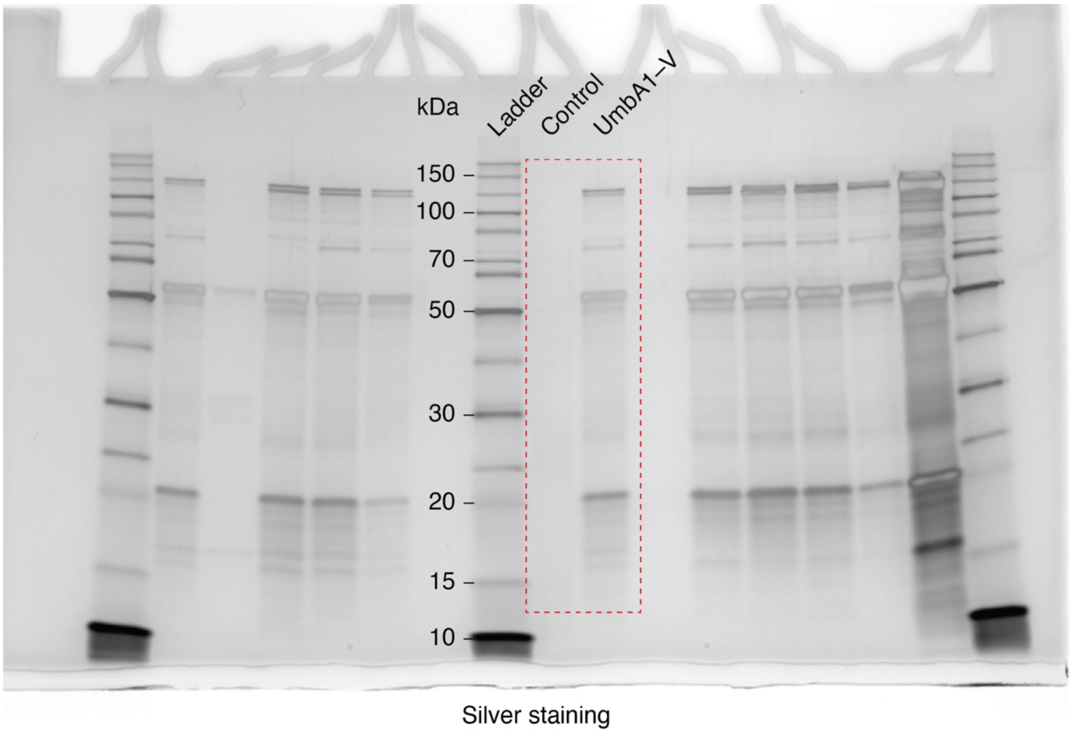

Uncropped blots, Fig. 3f

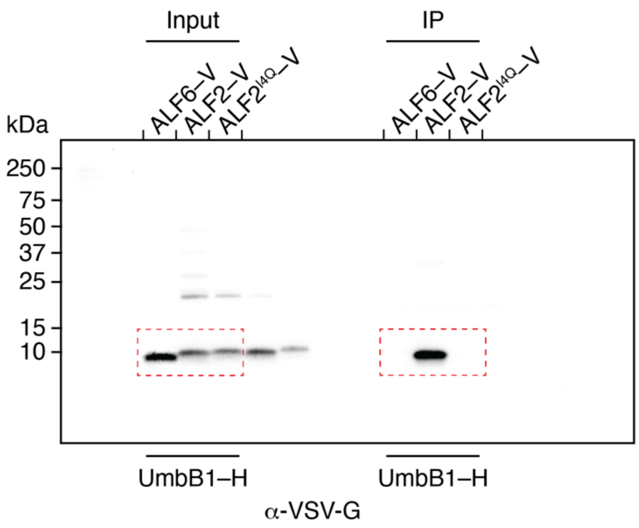

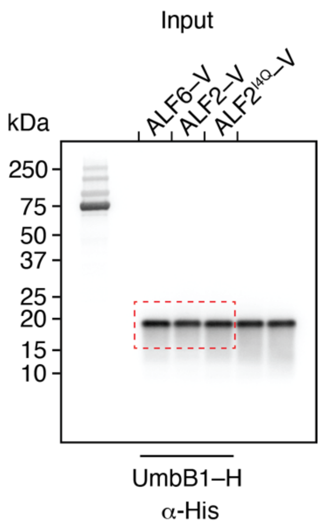

Uncropped blots, Extended Data Fig. 2

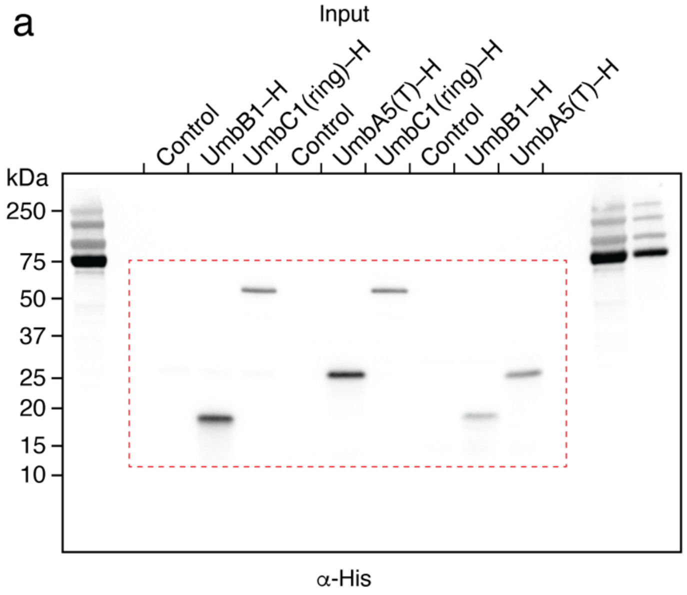

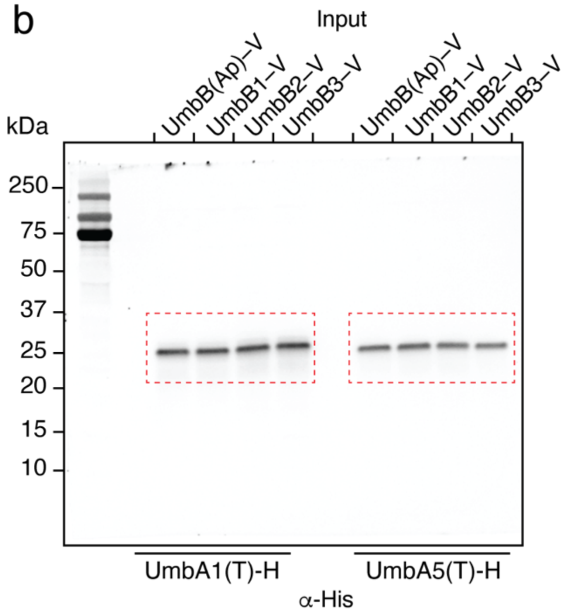

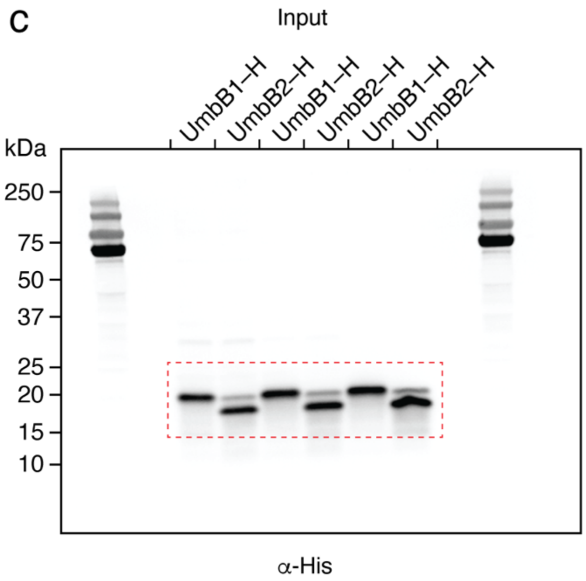

Uncropped blots, Extended Data Fig. 4a

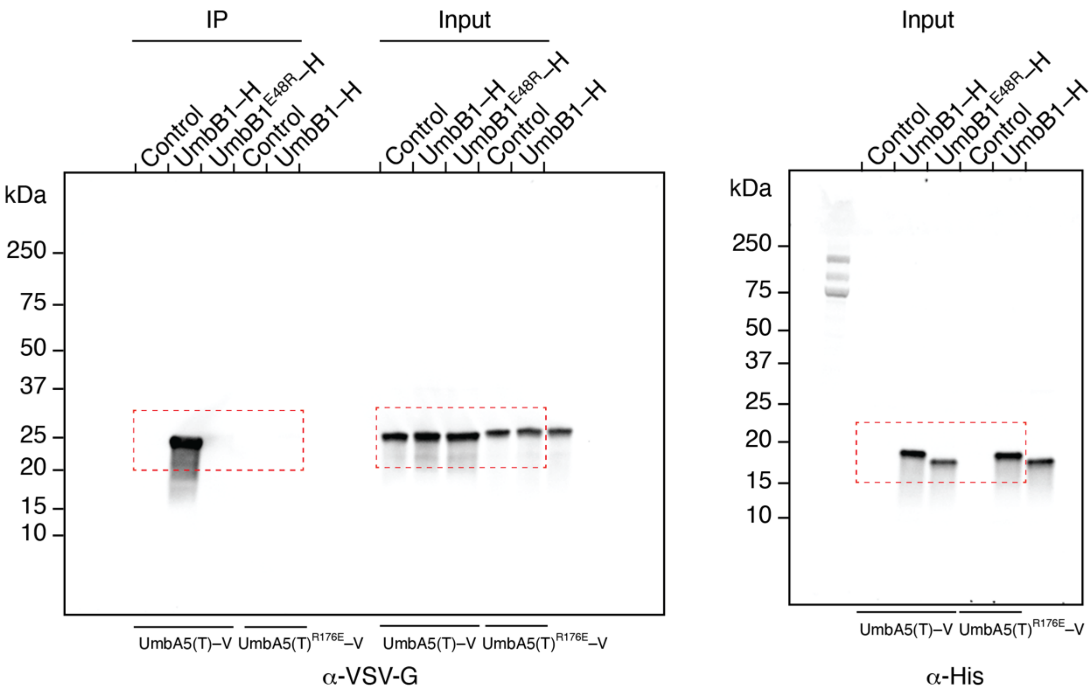

Uncropped gel, Extended Data Fig. 4c

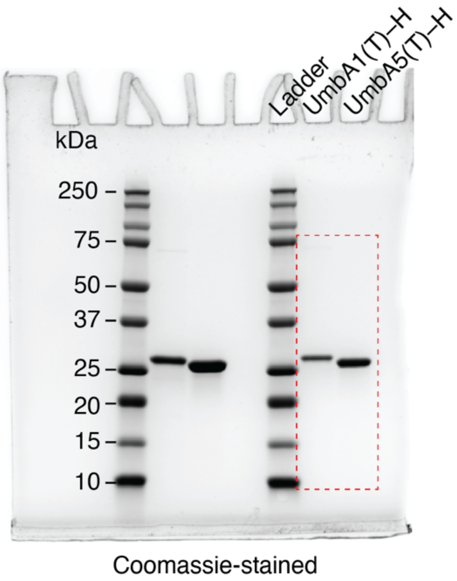

Uncropped gel, Extended Data Fig. 5b

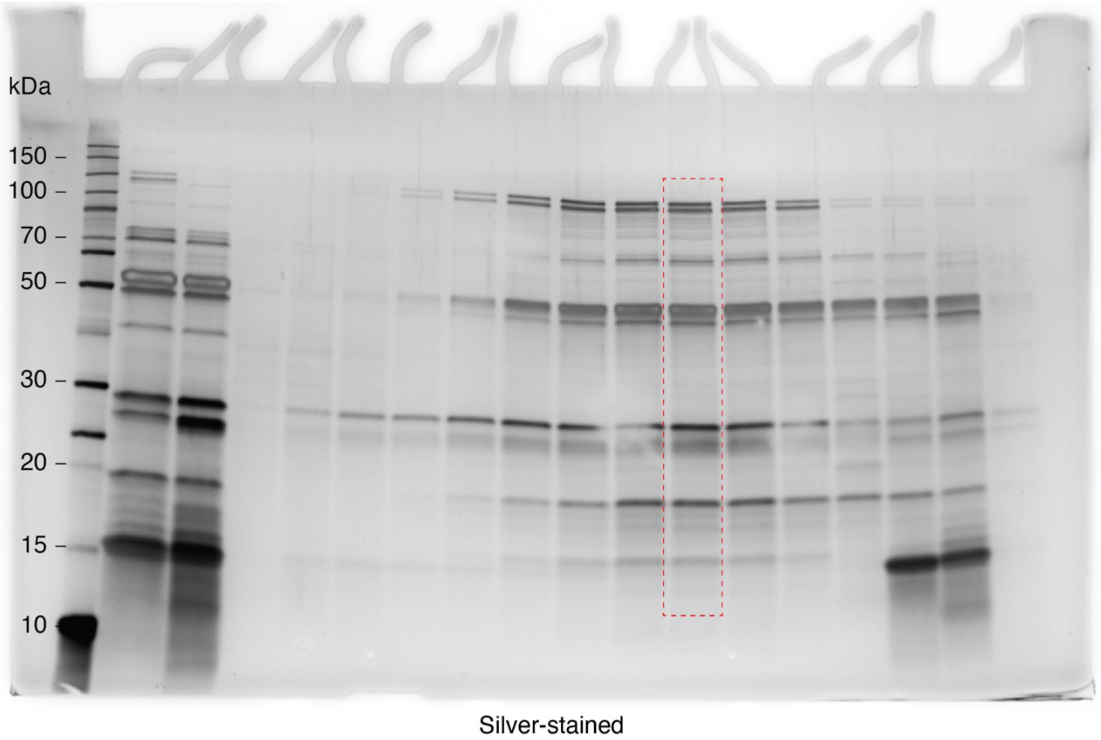

Supplementary Fig. 1: Uncropped gels and blots for data shown in main body and Extended Data figures. For each figure, the cropped regions are denoted by dashed boxes.

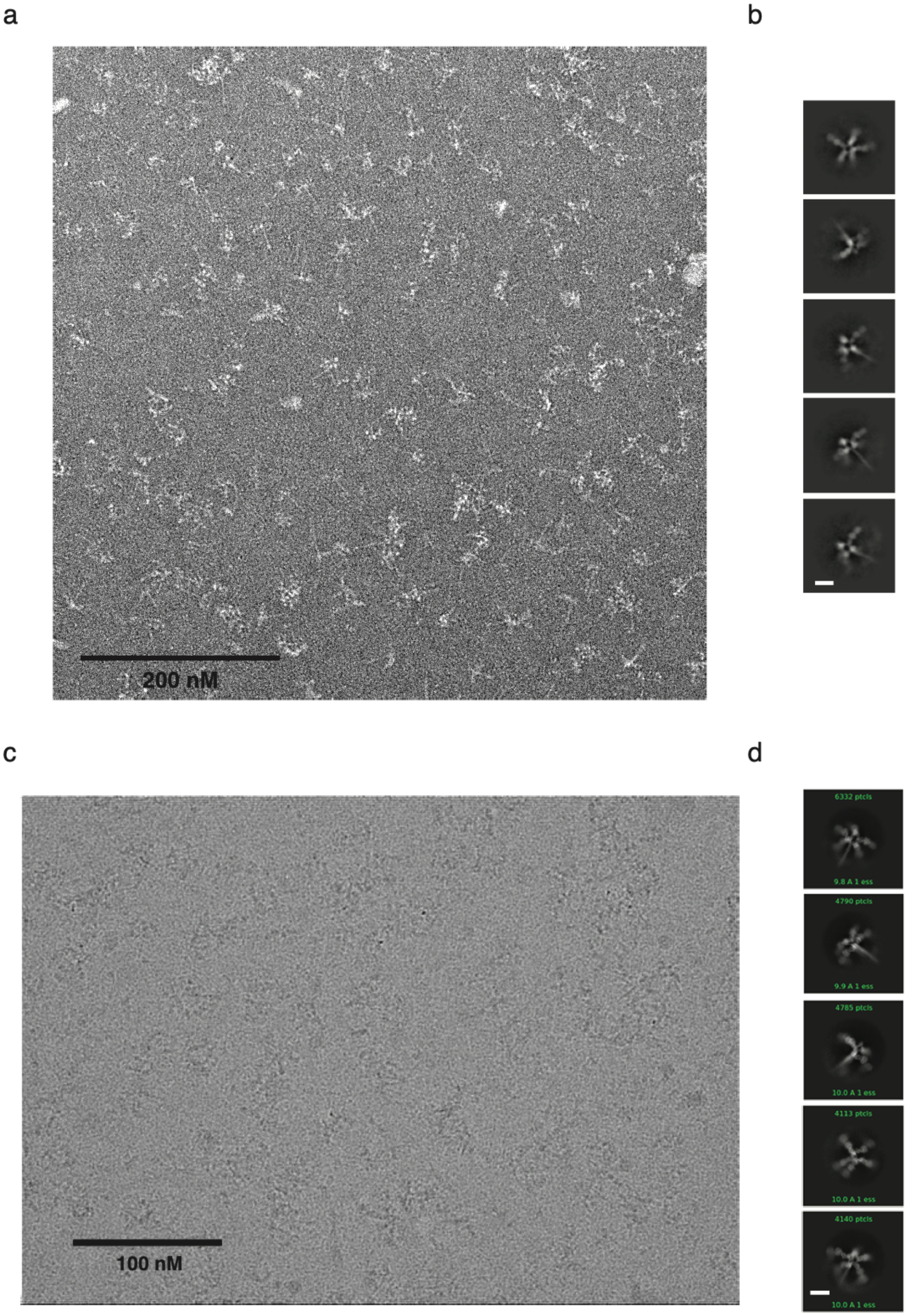

Supplementary Fig. 2: Full fields of view and 2D class averages of Umb1 particles from negative stain electron microscopy and cryoEM. a, b, Representative micrograph (a) and 2D class averages (b) from negative stain electron microscopy. Micrograph is representative of 230 collected. **c,d,** Represenative micrograph (c) and 2D class averages (d) from cryo-EM data collection. Micrograph in (c) is representative of 22,917 collected. Ptcls, number of particles represented; ess, effective sample size. Scale bar in (b) and (d), 100 Å.

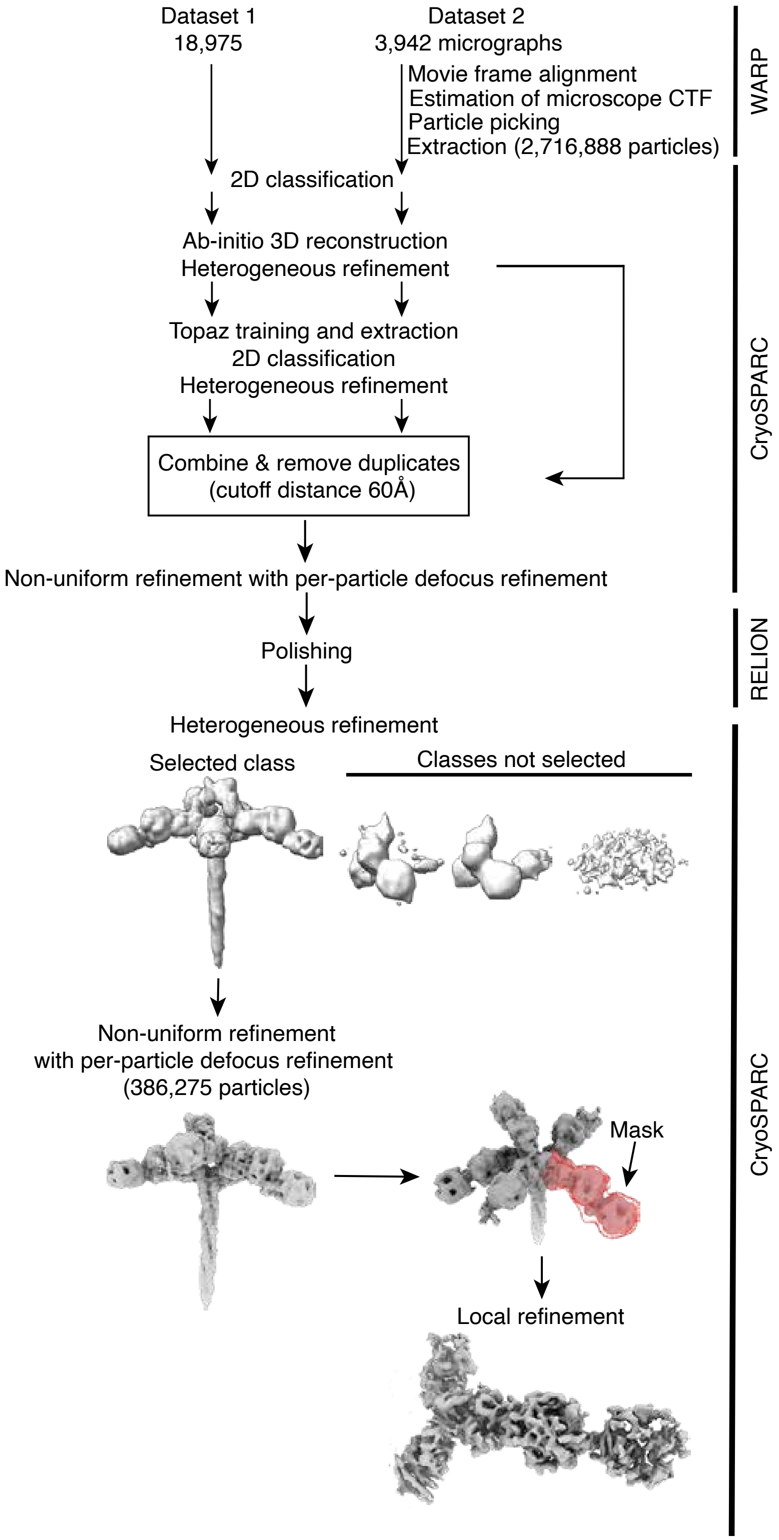

Supplementary Fig. 3: CryoEM Workflow. CryoEM data processing workflow for the Umb1 particle (details in methods section). CTF, contrast transfer function.

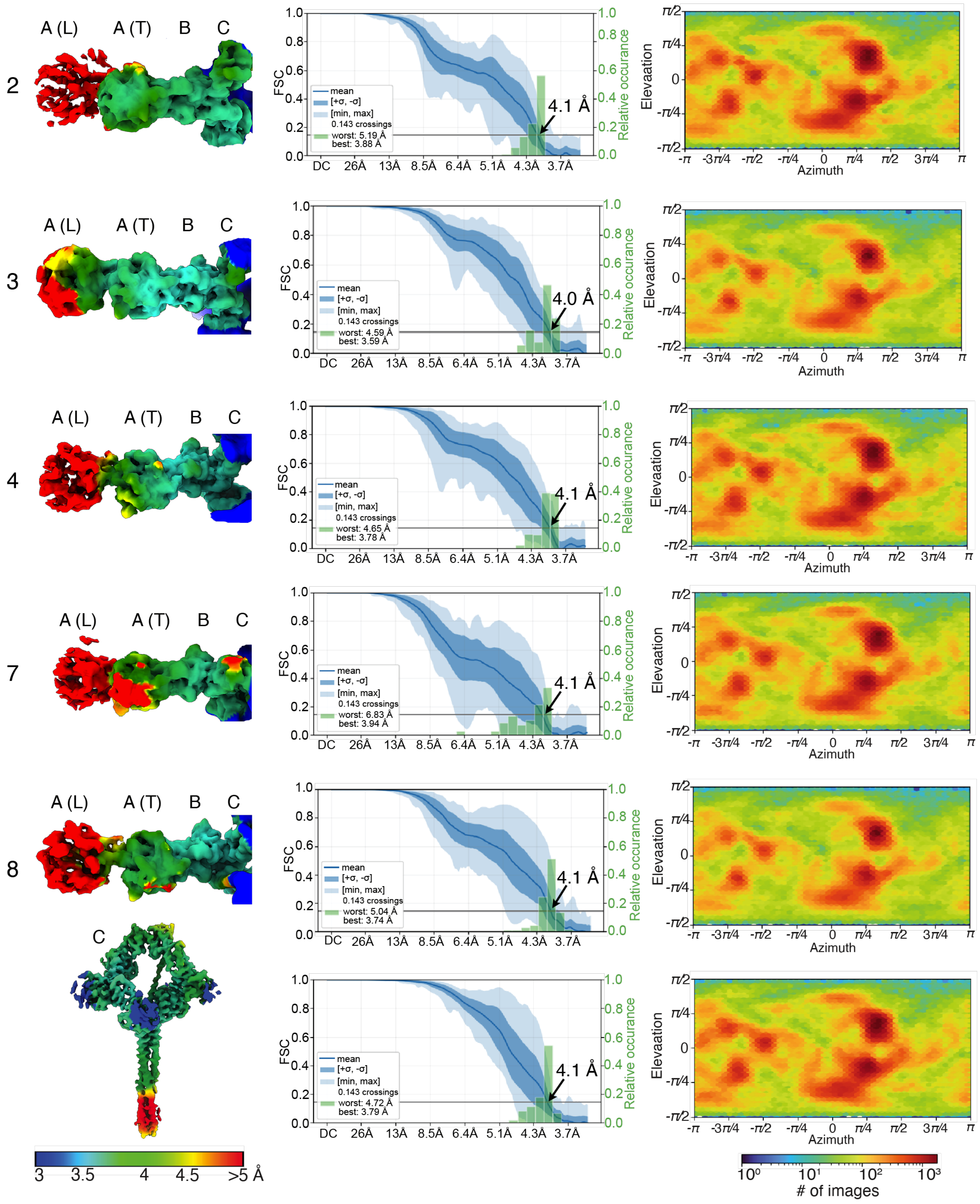

Supplementary Fig. 4. CryoEM Validation for the Umb1 spokes and stalk. Column 1: Local resolution maps of local refined maps encompassing each individual spoke, numbered to correspond with the interacting ALF repeat, and UmbC1. Column 2: FSC curves with the gold standard threshold of 0.143 and 3DFSC analysis for each spoke and UmbC1. Column 3: Particle distribution of local refined maps encompassing each spoke and UmbC1.

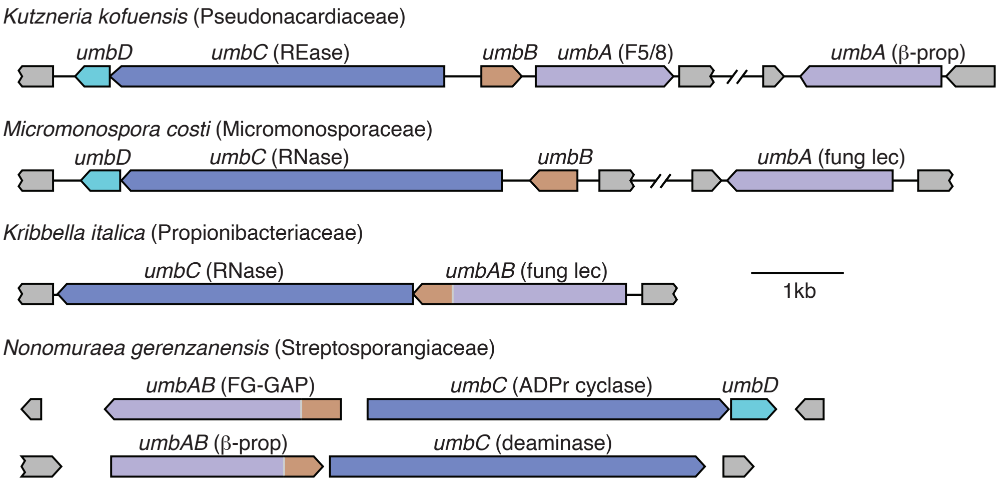

Supplementary Fig. 5. Representative *umb* loci from phylogenetically diverse Actinobacteria. Toxin domains encoded by the *umbC* genes and lectin domains encoded by the *umbA* genes are defined in Supplementary Tables 2 and 4, respectively. β-prop, UAL-βprop-1 family identified in this study.

Supplementary Tables.

Supplementary Table 3: Mass spectrometry-based identification of proteins immunoprecipitated by UmbC1, UmbC2, UmbC3 and UmbA1. Data were filtered to remove proteins with fewer than 6 spectral counts in the IP samples for both biological replicates, and only those proteins enriched at least log 2 = 0.5 are shown, with the exception of UmbA-VSV-G, for which the most-enriched non-Umb protein was included for context.

Bait protein	Protein ID	Control	IP	Enrichment (log2)
**UmbC1-VSV-G**				
	UmbA4	0	49	n/a
	UmbA5	0	40	n/a
	UmbD1	0	28.5	n/a
	UmbB1	0.5	18.5	n/a
	UmbA6	0	17	n/a
	UmbC1	1.5	134.5	6.02
	UmbA1	6.5	53	2.91
	Q8CJQ6	2	6.5	0.67
	Q8CK05	6.5	15.5	0.73
**UmbC2-VSV-G**				
	UmbC2	0.5	46.5	n/a
	UmbA4	0	12	n/a
	Q9L0Y3	1	11.5	n/a
	UmbA5	0.5	11	n/a
	UmbB2	0	9.5	n/a
	Q9KXP9	1.5	9.5	n/a
	UmbC1	0.5	7.5	n/a
	O54158	0.5	7	n/a
	UmbA1	1	15.5	3.61
	Q9RNU9	2.5	10.5	2.31
	Q9S274	1.5	8.5	2.22
	Q9XAC1	2	8	1.92
	Q9XA42	2	7	1.80
	Q9Z578	1.5	6	1.78
	O69998	3	6	1.40
	Q9FBR4	9.5	16.5	1.32
	UmbA1	4	11	1.25
	Q9RK35	3.5	9.5	1.25
	Q9KZP4	2.5	7.5	1.24
	Q9S2Q3|Q9FC62	6.5	13.5	1.10
	Q9L1Z5	3.5	8	1.08

	Q9XAP2	2.5	6.5	1.08
	Q9FC43	4	10	1.03
	Q9S2Q5|Q9FC63	6	9.5	1.01
	Q9XA23	5	7	1.01
	Q7AKF3	8.5	15.5	0.98
	Q9XAD1	7.5	18	0.94
	Q9KXR6	8	15.5	0.93
	Q9ZBU0	3.5	6.5	0.81
	Q9Z564	6	13	0.76
	Q9X909	8.5	14	0.67
	Q9ADJ6	3.5	6	0.66
	Q9EWV4	4.5	9	0.66
	Q9RKK5	8	16	0.62
	Q9L2H9	7.5	11	0.61
	Q93RV9	4	7.5	0.60
**UmbC3-VSV-G**				
	UmbA3	0.5	61.5	n/a
	UmbA4	0	45	n/a
	UmbB3	0	32	n/a
	UmbA6	0.5	26.5	n/a
	UmbA2	0	9	n/a
	UmbC3	10.5	781	4.71
	UmbA5	2	62	3.79
	UmbC1	1	9.5	1.70
	O70007	1.5	9.5	1.30
	Q9L244	2.5	8.5	0.92
**UmbA1-VSV-G**				
	UmbA4	0	51.5	n/a
	UmbA5	0	49.5	n/a
	UmbB1	0.5	38.5	n/a
	UmbA6	0	26	n/a
	UmbB2	0	11.5	n/a
	UmbC1	1.5	147.5	5.99
	UmbA1	6.5	145	4.41
	Q9X8D4	5	8.5	0.23

Supplementary Table 5: Growth conditions for and results from screening Umb supernatant for growth inhibitory activity against diverse bacteria. Growth was measured at a single timepoint using an ATP quantification-based bacterial cell viability assay. The average ratio (log 2) represents the viability of each strain grown with control supernatant divided by Umb supernatant treatment in two biological replicates of the screen. Z-scores were calculated from the average of log 2-transformed ratios from across all strains screened.

Phylum	Genus and species	Source	Media	Average ratio (log 2)	Z-score
Actinomycetota	*Allokutzneria albata*	NRRL No. B-24461	ISP2	0.281	0.296
Actinomycetota	*Amycolatopsis kentuckyensis* SAI_225	This study	TSBY	-1.175	-0.566
Actinomycetota	*Amycolatopsis sp.* SAI_101	This study	TSBY	1.060	0.757
Actinomycetota	*Arthrobacter nitroguajacolicus* SAI_060	This study	TSBY	-0.584	-0.217
Actinomycetota	*Arthrobacter oxydans* SAI_217	This study	TSBY	-0.956	-0.436
Actinomycetota	*Brevibacterium lyticum*	NRRL No. B-4262	TGY	-0.356	-0.081
Actinomycetota	*Catenulispora acidophila*	NRRL No. B-24433	ISP2	-0.359	-0.083
Actinomycetota	*Cellulosimicrobium cellulans*	NRRL No. B-2381	TGY	-0.990	-0.457
Actinomycetota	*Cellulosimicrobium cellulans* SAI_258	This study	TSBY	-1.093	-0.517
Actinomycetota	*Cellulosimicrobium sp.* SAI_197	This study	TSBY	-1.254	-0.613
Actinomycetota	*Dermacoccus sp.* SAI_028	This study	TSBY	0.289	0.301
Actinomycetota	*Dermatophilus congolensis*	NRRL No. B-2350	TSBY	-1.626	-0.833
Actinomycetota	*Frankia sp.*	NRRL No. B-16219	ISP2	0.390	0.360
Actinomycetota	*Georgenia sp.*	NRRL No. B-59275	TGY	-1.048	-0.491
Actinomycetota	*Glycomyces rutgersensis*	NRRL No. B-16106	ISP2	-2.024	-1.069
Actinomycetota	*Kibdelosporangium aridum*	NRRL No. B-16436	ISP2	0.135	0.210
Actinomycetota	*Kineococcus gynurae*	NRRL No. B-24568	ISP2	-2.100	-1.114
Actinomycetota	*Kitasatospora kifunensis*	NRRL No. B-24284	LB	2.144	1.399
Actinomycetota	*Kribbella hippodromi*	NRRL No. B-24553	ISP2	-0.226	-0.004
Actinomycetota	*Leifsonia sp.* 004_C5	This study	TSBY	0.491	0.420
Actinomycetota	*Leifsonia sp.* 006_B1	This study	TSBY	-0.166	0.031
Actinomycetota	*Microbacterium atlanticum* SAI_030	This study	TSBY	-0.333	-0.067
Actinomycetota	*Microbacterium jejuense* SAI_031	This study	TSBY	-2.428	-1.308
Actinomycetota	*Microbacterium paraoxydans* SAI_221	This study	TSBY	-1.434	-0.720
Actinomycetota	*Microbacterium trichothecenolyticum* CFG_272	This study	TSBY	-0.706	-0.288
Actinomycetota	*Microtetraspora glauca*	NRRL No. B-3735	ISP2	-0.026	0.114
Actinomycetota	*Mycobacterium goodii* 002_G6	This study	TSBY	0.985	0.713
Actinomycetota	*Mycobacterium smegmatis* 001_A6	This study	TSBY	-1.658	-0.852
Actinomycetota	*Nocardioides luteus*	NRRL No. B-16231	R2A	0.313	0.315

Actinomycetota	*Nocardioides sp.* HB12	PMID:29556109	R2A	-0.228	-0.006
Actinomycetota	*Nocardioides sp.* SAI_065	This study	TSBY	-0.419	-0.119
Actinomycetota	*Oerskovia turbata*	NRRL No. B-8019	ISP2	-0.001	0.129
Actinomycetota	*Patulibacter minatonensis*	NRRL No. B-24346	ISP2	-0.799	-0.343
Actinomycetota	*Promicromonospora kroppenstedtii* TBS_116	This study	TSBY	0.188	0.241
Actinomycetota	*S. albogriseolus* SAI_173	This study	TSBY	0.705	0.547
Actinomycetota	*S. albogriseolus* SAI_190	This study	TSBY	-0.210	0.005
Actinomycetota	*S. ambofaciens*	NRRL No. B-2516	TSBY	-0.633	-0.245
Actinomycetota	*S. ambofaciens* SAI_080	This study	TSBY	8.434	5.124
Actinomycetota	*S. ambofaciens* SAI_104	This study	TSBY	3.839	2.403
Actinomycetota	*S. ambofaciens* SAI_108	This study	TSBY	-1.135	-0.542
Actinomycetota	*S. ambofaciens* SAI_195	This study	TSBY	10.827	6.541
Actinomycetota	*S. antibioticus*	NRRL No. B-1701	TSBY	-1.104	-0.524
Actinomycetota	*S. anulatus*	NRRL No. B-2000	TSBY	-0.114	0.062
Actinomycetota	*S. azureus*	NRRL No. B-2655	TSBY	-0.187	0.019
Actinomycetota	*S. cellulosae* SAI_051	This study	TSBY	-0.683	-0.275
Actinomycetota	*S. coelicolor*	NRRL No. B-3062	TSBY	-0.171	0.029
Actinomycetota	*S. collinus* SAI_078	This study	TSBY	3.028	1.923
Actinomycetota	*S. corchorusii* SAI_180	This study	TSBY	0.344	0.333
Actinomycetota	*S. eurocidicus*	NRRL No. B-1701	TSBY	0.286	0.299
Actinomycetota	*S. graminofaciens* SAI_110	This study	TSBY	-0.130	0.052
Actinomycetota	*S. graminofaciens* SAI_175	This study	TSBY	-0.331	-0.067
Actinomycetota	*S. graminofaciens* SAI_211	This study	TSBY	0.091	0.184
Actinomycetota	*S. griseorubiginosus* SAI_142	This study	TSBY	-0.212	0.004
Actinomycetota	*S. griseus*	NRRL No. B-2682	TSBY	4.665	2.892
Actinomycetota	*S. lividans*	NRRL No. B65306	TSBY	-0.045	0.103
Actinomycetota	*S. luteogriseus*	NRRL No. B-12422	TSBY	2.698	1.727
Actinomycetota	*S. mobaraensis*	NRRL No. B-3729	TSBY	0.358	0.342
Actinomycetota	*S. pristinaespiralis*	NRRL No. B2958	TSBY	-0.010	0.124
Actinomycetota	*S. pristinaespiralis* SAI_178	This study	TSBY	-0.662	-0.263
Actinomycetota	*S. rochei* SAI_164	This study	TSBY	2.083	1.363
Actinomycetota	*S. sp.* SAI_041	This study	TSBY	-0.573	-0.210
Actinomycetota	*S. sp.* SAI_103	This study	TSBY	2.574	1.654
Actinomycetota	*S. sp.* SAI_117	This study	TSBY	-0.264	-0.027
Actinomycetota	*S. sp.* SAI_126	This study	TSBY	-0.238	-0.011
Actinomycetota	*S. sp.* SAI_133	This study	TSBY	-0.074	0.085
Actinomycetota	*S. sp.* SAI_167	This study	TSBY	1.935	1.275
Actinomycetota	*S. sp.* SAI_203	This study	TSBY	-0.343	-0.074

Actinomycetota	*S. tendae* SAI_182	This study	TSBY	-1.268	-0.621
Actinomycetota	*S. tendae* SAI_185	This study	TSBY	-0.274	-0.033
Actinomycetota	*S. tendae* SAI_218	This study	TSBY	-0.306	-0.051
Actinomycetota	*Saccharothrix coeruleofusca*	NRRL No. B-16115	LB	2.528	1.626
Actinomycetota	*Sanguibacter sp.*	NRRL No. B-59339	TGY	-0.769	-0.326
Actinomycetota	*Terrabacter tumescens*	NRRL No. B-4012	TGY	-0.997	-0.461
Bacteroidota	*Chryseobacterium bacterium* CI02	PMID:16885294	TSBY	-0.591	-0.220
Bacteroidota	*Flavobacterium johnsoniae* UW101	Gift from Mark McBride's lab	LB	-0.328	-0.065
Bacteroidota	*Sphingobacterium bacterium* CI01	PMID:16885294	TSBY	-0.409	-0.112
Firmicutes	*Alkalihalobacillus alcalophilus*	NRRL No. B-14309	Alkaline Nutrient Agar	0.005	0.132
Firmicutes	*Bacillus aryabhattai* TBS_067	This study	TSBY	-1.011	-0.469
Firmicutes	*Bacillus haynesii* TBS_113	This study	TSBY	-0.821	-0.356
Firmicutes	*Bacillus megaterium* 005_H9	This study	TSBY	-0.725	-0.300
Firmicutes	*Bacillus pumilus* TBS_051	This study	TSBY	-0.994	-0.459
Firmicutes	*Bacillus pumilus* TBS_099	This study	TSBY	-0.873	-0.388
Firmicutes	*Bacillus sp.* 005_H12	This study	TSBY	-0.887	-0.396
Firmicutes	*Bacillus sp.* 006_D12	This study	TSBY	-1.045	-0.489
Firmicutes	*Bacillus subtilis* 004_F4	This study	TSBY	-0.655	-0.258
Firmicutes	*Bacillus subtilis* 006_C4	This study	TSBY	-0.998	-0.462
Firmicutes	*Bacillus velezensis* 006_A12	This study	TSBY	-0.598	-0.224
Firmicutes	*Bacillus zanthoxyli* TBS_040	This study	TSBY	1.018	0.732
Firmicutes	*Bacillus zanthoxyli* TBS_056	This study	TSBY	-0.804	-0.346
Firmicutes	*Brevibacillus borstelensis*	NRRL No. NRS-948	TGY	-1.339	-0.663
Firmicutes	*Brevibacillus chosinensis*	NRRL No. B-23247	TGY	-0.923	-0.417
Firmicutes	*Brevibacillus laterosporus*	NRRL No. NRS-1339	TGY	-1.317	-0.650
Firmicutes	*Brevibacillus parabrevis*	NRRL No. NRS-751	TGY	-1.124	-0.536
Firmicutes	*Carnobacterium divergens*	NRRL No. B-23835	Liver Infusion Broth	-0.850	-0.374
Firmicutes	*Carnobacterium gallinarum*	NRRL No. B-14832	Liver Infusion Broth	-1.293	-0.636
Firmicutes	*Carnobacterium maltaromaticum*	NRRL No. B-14829	Liver Infusion Broth	-1.441	-0.724
Firmicutes	*Caryophanon latum*	NRRL No. B-1893	ISP2	-0.878	-0.390
Firmicutes	*Cytobacillus kochii*	NRRL No. NRS-1758	TGY	-1.578	-0.805
Firmicutes	*Cytobacillus praedii*	NRRL No. B-14566	TGY	-1.151	-0.552
Firmicutes	*Fictibacillus marinisediminis*	NRRL No. B-59209	TGY	0.091	0.184
Firmicutes	*Gracibacillus dipsosauri*	NRRL No. B-23348	TSBY	-0.765	-0.323
Firmicutes	*Heyndrickxia sporothermodurnas*	NRRL No. NRS-1638	TGY	-0.872	-0.387
Firmicutes	*Kurthia gibsonii*	NRRL No. B-41085	TGY	-2.253	-1.205
Firmicutes	*Kurthia sibirica*	NRRL No. B-41083	TGY	-0.691	-0.279

Firmicutes	*Lactococcus lactis*	NRRL No. B-23804	Liver Infusion Broth	-1.762	-0.914
Firmicutes	*Leuconostoc citreum*	NRRL No. B-1501	Liver Infusion Broth	-1.138	-0.544
Firmicutes	*Lysinibacillus fusiformis*	NRRL No. B-14865	TGY	-1.121	-0.534
Firmicutes	*Lysinibacillus xylanityticus*	NRRL No. NRS-1307	TGY	-1.037	-0.485
Firmicutes	*Neobacillus mesonae*	NRRL No. B-14565	TGY	-0.760	-0.320
Firmicutes	*Oenococcus oeni*	NRRL No. B-3474	Liver Infusion Broth	-0.961	-0.439
Firmicutes	*Paenibacillus alba*	NRRL No. BD-533	TGY	-0.155	0.038
Firmicutes	*Paenibacillus amylolyticus*	NRRL No. B-14940	TGY	0.342	0.332
Firmicutes	*Paenibacillus chibensis*	NRRL No. B-142	TGY	-1.367	-0.680
Firmicutes	*Paenibacillus favisporus* 004_C2	This study	TSBY	-0.740	-0.308
Firmicutes	*Paenibacillus lautus* 004_C1	This study	TSBY	-0.816	-0.354
Firmicutes	*Paenibacillus lautus* TBS_091	This study	TSBY	0.067	0.169
Firmicutes	*Paenibacillus lautus* TBS_092	This study	TSBY	-2.070	-1.096
Firmicutes	*Pediococcus pentosaceus*	NRRL No. B-14620	Liver Infusion Broth	-1.343	-0.666
Firmicutes	*Peribacillus castrilensis*	NRRL No. B-41276	TGY	-1.832	-0.955
Firmicutes	*Peribacillus frigoritolerans*	NRRL No. BD-432	TGY	-0.808	-0.349
Firmicutes	*Peribacillus simplex*	NRRL No. BD-267	TGY	-2.190	-1.167
Firmicutes	*Planococcus sierraensis*	NRRL No. B-65582	R2A	-0.690	-0.279
Firmicutes	*Priestia aryabhattai*	NRRL No. BD-263	TGY	0.703	0.546
Firmicutes	*Priestia endophytica*	NRRL No. BD-290	TGY	-0.665	-0.264
Firmicutes	*Rummeliibacillus stabekisii*	NRRL No. B-51320	TSBY	-1.180	-0.569
Firmicutes	*Sporosarcina globispora*	NRRL No. B-3396	TGY	-0.273	-0.032
Firmicutes	*Staphylococcus sp.* HA57	PMID:29556109	R2A	-0.799	-0.343
Firmicutes	*Sutcliffiella cohnii*	NRRL No. B-14735	Alkaline Nutrient Agar	-1.104	-0.524
Firmicutes	*Weissella confusa*	NRRL No. B-1064	Liver Infusion Broth	-1.089	-0.515
Firmicutes	*Weissella viridescens*	NRRL No. B-1951	Liver Infusion Broth	-1.472	-0.742
Proteobacteria	*Acinetobacter baylyi* ADP1	ATCC #33305	BHI	-0.644	-0.252
Proteobacteria	*Agrobacterium tumefaciens* FACH	Dong *et al.* 1992. *Phytopathology.*	TSBY	-0.305	-0.051
Proteobacteria	*Burkholderia thailandensis* E264	PMID:16725056	LB	0.175	0.233
Proteobacteria	*Erwinia caratovora* Ecc71	PMID:9701816	LB	-0.074	0.086
Proteobacteria	*Escherichia coli* MG1655	ATCC #47076	LB	-1.006	-0.466
Proteobacteria	*Lysobacter enzymogenes* UASM495	ATCC #29487	TSBY	-0.109	0.065
Proteobacteria	*Pseudomonas aeruginosa* PAO1	PMID:10984043	LB	-0.442	-0.132
Proteobacteria	*Serratia proteamaculans*	PMID:26187596	LB	-0.255	-0.021
Proteobacteria	*Xanthomonas maltophilia*	ATCC #13637	TSBY	-0.579	-0.213
