# Supplementary material for: Streptomyces umbrella toxin particles block hyphal growth of competing species
Source: Nature. 2024 Apr 17;629(8010):165–73. doi: 10.1038/s41586-024-07298-z (PMC11062931; doi:10.1038/s41586-024-07298-z)
Supplement: Supplementary file 2 — Reporting Summary [file 41586_2024_7298_MOESM2_ESM.pdf]

nature portfolio

Corresponding author(s): Joseph Mougous

Last updated by author(s): Feb 23, 2024

Reporting Summary

Nature Portfolio wishes to improve the reproducibility of the work that we publish. This form provides structure for consistency and transparency in reporting. For further information on Nature Portfolio policies, see our Editorial Policies and the Editorial Policy Checklist.

Statistics

For all statistical analyses, confirm that the following items are present in the figure legend, table legend, main text, or Methods section.

n/a	Confirmed	
☐	☒	The exact sample size (*n*) for each experimental group/condition, given as a discrete number and unit of measurement
☐	☒	A statement on whether measurements were taken from distinct samples or whether the same sample was measured repeatedly
☐	☒	The statistical test(s) used AND whether they are one- or two-sided<br>*Only common tests should be described solely by name; describe more complex techniques in the Methods section.*
☒	☐	A description of all covariates tested
☒	☐	A description of any assumptions or corrections, such as tests of normality and adjustment for multiple comparisons
☐	☒	A full description of the statistical parameters including central tendency (e.g. means) or other basic estimates (e.g. regression coefficient) AND variation (e.g. standard deviation) or associated estimates of uncertainty (e.g. confidence intervals)
☐	☒	For null hypothesis testing, the test statistic (e.g. *F*, *t*, *r*) with confidence intervals, effect sizes, degrees of freedom and *P* value noted<br>*Give P values as exact values whenever suitable.*
☒	☐	For Bayesian analysis, information on the choice of priors and Markov chain Monte Carlo settings
☒	☐	For hierarchical and complex designs, identification of the appropriate level for tests and full reporting of outcomes
☒	☐	Estimates of effect sizes (e.g. Cohen's *d*, Pearson's *r*), indicating how they were calculated

Our web collection on statistics for biologists contains articles on many of the points above.

Software and code

Policy information about availability of computer code

Data collection	CryoEM and negative stain EM data collection were carried out using Leginon v3.5. Fluorescence and phase-contrast microscopy data was collected using NIS-Elements v3.30.02.
Data analysis	Negative stain EM data processing: CTFFIND v4, DoGPicker v1, Relion v3.0, CryoSPARC v4.4.0<br>CryoEM data processing: Warp v1.0.0, CryoSPARC v4.4.0, Relion 3.1 v3.1, pyem v0.5<br>Protein structure modeling and visualization: AlphaFold2, RoseTTAFold2 v2020.08.61146, ColabFold, ChimeraX v1.6.1<br>Fluorescence and phase-contrast microscopy data analysis: Omnipose 1.0.6, FIJI 2.14.0, Napari 0.4.18, Python 3.10.9<br>Statistical analysis of bacterial growth assays: GraphPad Prism v10.1.1<br>Bioinformatic analysis: PSI-BLAST 2.13.0+, BLASTCLUST 2.2.26, HMMSCAN (HMMER 3.3.2 package), Phobius v1.01, CLANS, Fruchterman and Reingold force-directed layout algorithm, KALIGN v3.3.2, MUSCLE v3.8.1551, PROMALS3D, Chroma 1.0, DALI v0.99.95, Foldseek, hhblits, Geneious Prime 2023.2.1

For manuscripts utilizing custom algorithms or software that are central to the research but not yet described in published literature, software must be made available to editors and reviewers. We strongly encourage code deposition in a community repository (e.g. GitHub). See the Nature Portfolio guidelines for submitting code & software for further information.

Data

Policy information about availability of data

All manuscripts must include a data availability statement. This statement should provide the following information, where applicable:

- Accession codes, unique identifiers, or web links for publicly available datasets
- A description of any restrictions on data availability
- For clinical datasets or third party data, please ensure that the statement adheres to our policy

All source data for this study will be provided with this paper or are deposited in a public repository. The cryo-EM maps and atomic structures are under deposition in the Protein Data Bank (PDB) and/or Electron Microscopy Data Bank (EMDB) under accession codes PDB ID 8W20, PDB ID 8W22, EMD-43736 and EMD-43737. Bacterial protein sequences used for assessing the diversity and distribution of Umb toxins were obtained from the NCBI non-redundant (nr) protein database.

Research involving human participants, their data, or biological material

Policy information about studies with human participants or human data. See also policy information about sex, gender (identity/presentation), and sexual orientation and race, ethnicity and racism.

Reporting on sex and gender	Not applicable.
Reporting on race, ethnicity, or other socially relevant groupings	Not applicable.
Population characteristics	Not applicable.
Recruitment	Not applicable.
Ethics oversight	Not applicable.

Note that full information on the approval of the study protocol must also be provided in the manuscript.

Field-specific reporting

Please select the one below that is the best fit for your research. If you are not sure, read the appropriate sections before making your selection.

☒ Life sciences ☐ Behavioural & social sciences ☐ Ecological, evolutionary & environmental sciences

For a reference copy of the document with all sections, see nature.com/documents/nr-reporting-summary-flat.pdf

Life sciences study design

All studies must disclose on these points even when the disclosure is negative.

Sample size	Sample size was not statistically predetermined. For experiments in which statistical analysis was applied, a minimum of three replicates were performed to ensure validity of the analyses. For Western blot analyses, a minimum of two biological replicates were performed as is standard in the field, see for example https://www.nature.com/articles/s41586-023-06506-6#MOESM2. For the cryo-EM experiment, data were collected until the resolution could no longer be improved through further data acquisition.
Data exclusions	Proteins with low spectral counts (<6) in IP samples were excluded from the enrichment analysis.
Replication	Experiments were independently replicated a minimum of two times, as described in figure legends. All attempts at replication were successful.
Randomization	This was not relevant to our study, as experiments were performed using clonal bacterial populations or were in vitro assays of purified proteins or lysates of clonal populations of bacteria.
Blinding	Blinding was not relevant to our study, as subjective analysis of the data was not required.

Reporting for specific materials, systems and methods

We require information from authors about some types of materials, experimental systems and methods used in many studies. Here, indicate whether each material, system or method listed is relevant to your study. If you are not sure if a list item applies to your research, read the appropriate section before selecting a response.

Materials & experimental systems

n/a	Involved in the study	
☐	☒	Antibodies
☒	☐	Eukaryotic cell lines
☒	☐	Palaeontology and archaeology
☒	☐	Animals and other organisms
☒	☐	Clinical data
☒	☐	Dual use research of concern
☒	☐	Plants

Methods

n/a	Involved in the study	
☒	☐	ChIP-seq
☒	☐	Flow cytometry
☒	☐	MRI-based neuroimaging

Antibodies

Antibodies used	anti-VSV-G Sigma, V4888; anti-His HRP conjugated, Qiagen 34460; anti-rabbit HRP conjugated, Sigma A6154
Validation	All antibodies are commercially available. Validation information is provided in datasheets supplied by the manufacturers. See https://www.sigmaaldrich.com/deepweb/assets/sigmaaldrich/product/documents/343/088/v4888dat.pdf for anti-VSV-G and https://www.qiagen.com/us/knowledge-and-support/product-and-technical-support/quality-and-safety-data/sds-search?l=US&q=800000000487 for anti-His HRP conjugated.

Plants

Seed stocks	Not applicable.
Novel plant genotypes	Not applicable.
Authentication	Not applicable.
